# Comparative genomics reveals diversified CRISPR-Cas systems of globally distributed *Microcystis aeruginosa*, a freshwater bloom-forming cyanobacterium

**DOI:** 10.3389/fmicb.2015.00394

**Published:** 2015-05-12

**Authors:** Chen Yang, Feibi Lin, Qi Li, Tao Li, Jindong Zhao

**Affiliations:** ^1^Key Laboratory of Algal Biology, Institute of Hydrobiology, Chinese Academy of ScienceWuhan, China; ^2^University of Chinese Academy of SciencesBeijing, China; ^3^College of Life Science, Peking UniversityBeijing, China

**Keywords:** comparative genomics, *Microcystis aeruginosa*, CRISPR-Cas system, harmful algal blooms, freshwater cyanobacterium

## Abstract

*Microcystis aeruginosa* is one of the most common and dominant bloom-forming cyanobacteria in freshwater lakes around the world. *Microcystis* cells can produce toxic secondary metabolites, such as microcystins, which are harmful to human health. Two *M. aeruginosa* strains were isolated from two highly eutrophic lakes in China and their genomes were sequenced. Comparative genomic analysis was performed with the 12 other available *M. aeruginosa* genomes and closely related unicellular cyanobacterium. Each genome of *M. aeruginosa* containing at least one clustered regularly interspaced short palindromic repeat (CRISPR) locus and total 71 loci were identified, suggesting it is ubiquitous in *M. aeruginosa* genomes. In addition to the previously reported subtype I-D *cas* gene sets, three CAS subtypes I-A, III-A and III-B were identified and characterized in this study. Seven types of CRISPR direct repeat have close association with CAS subtype, confirming that different and specific secondary structures of CRISPR repeats are important for the recognition, binding and process of corresponding *cas* gene sets. Homology search of the CRISPR spacer sequences provides a history of not only resistance to bacteriophages and plasmids known to be associated with *M. aeruginosa*, but also the ability to target much more exogenous genetic material in the natural environment. These adaptive and heritable defense mechanisms play a vital role in keeping genomic stability and self-maintenance by restriction of horizontal gene transfer. Maintaining genomic stability and modulating genomic plasticity are both important evolutionary strategies for *M. aeruginosa* in adaptation and survival in various habitats.

## Introduction

Water eutrophication has become a major environmental problem all over the world as it induces the expansion and persistence of Harmful Algal Blooms (HABs) (Heisler et al., 2008; Smith and Schindler, 2009). HABs include different algal taxa such as dinoflagellates, diatoms and cyanobacterium. Cyanobacterium, known as blue-green algae, are of special concern because they grow photoautotrophically and migrate rapidly, floating on the surface or subsurface water, stopping sunlight from reaching other photosynthetic plants and causing hypoxia in the water body. *Microcystis* species are dominant freshwater bloom-forming cyanobacterium and produce a range of toxic organic compounds that can affect human and animal health through drinking and recreational water (Paerl et al., 2001; Westrick et al., 2010). Of this genus, *M. aeruginosa* is the most typical and notorious species, mainly because of the production of microcystins, which have been the chief agent in numerous cases of animal and human poisoning (Briand et al., 2003; Soares et al., 2006).

The genetic background of *Microcystis* was barely known until the complete genome sequence of *M. aeruginosa* NIES-843 was published in 2007 (Kaneko et al., 2007), followed by that of strain PCC 7806 in 2008 (Frangeul et al., 2008). Both genomes show high plasticity, with ~11.7% repeat sequence comprised of insertion sequences and transposable elements. In addition to multiple gene clusters involved in synthesis of secondary metabolites in both genomes, many genes for restriction modification (R-M) systems were also identified. Not all *M. aeruginosa* strains are toxin-producing. Toxic and non-toxic strains usually co-exist in a water body and it is hard to distinguish them under a microscope (Rantala et al., 2006). Genome comparison between non-toxic and toxic strains of *M. aeruginosa* revealed that they share less than half of their genes, and suggested numerous genes had been gained during evolution (Yang et al., 2013).

Mobile Genetic elements (MGEs) including bacteriophages, plasmids are found abundant in marine and freshwater environments (Miller and Capy, 2004). Horizontal transfer involving MGEs is a key force driving bacterial evolution (Goodier and Kazazian, 2008; Koonin and Wolf, 2008) and it is much more frequent than previously realized (Mcdaniel et al., 2010). In response, bacteria have developed several defense mechanisms such as uptake block, abortive infection, restriction-modification (R-M) system and CRISPR-Cas system (Westra et al., 2012) to restrict it. Among these, R-M system is possessed by most bacteria and archaea (Labrie et al., 2010), which mainly encode restriction endonucleases (REase) and methyltransferases. REases cleave DNA at specific sites, while MTases modify a particular sequence to protect it from REase cleavage (Mruk and Kobayashi, 2013). There are four classical groups of R-M systems with differences in molecular structure, sequence recognition, cleavage positions and cofactor requirements (Roberts et al., 2003).

CRISPR-Cas systems are composed of the CRISPR array and the CRISPR associated genes (*cas*). CRISPR (clustered regularly interspaced short palindromic repeats) loci are short direct repeats (DRs) separated by non-repetitive sequences (spacers), widely distributed in the majority of archaeal and approximately half of bacterial genomes (Grissa et al., 2007a; Sorek et al., 2008). Depending on species, DRs range from 24 to 48 bp while spacers range from 26 to 72 bp in length (Grissa et al., 2007b). DRs are usually identical within a locus, whereas spacers originating from foreign bacteriophages and plasmids are often unique, even among strains of same species (Pourcel et al., 2005). CRISPR transcripts are processed into small interfering RNAs that guide a multifunctional protein complex to recognize and cleave matching foreign DNA. The palindromic signature of DRs is thought to be indicative of a functional RNA secondary structure (Kunin et al., 2007). CRISPR associated genes (*cas*) are encoding a large and heterogeneous family of proteins that carry functional domains typical of nucleases, helicases, polymerases and polynucleotide-binding proteins (Haft et al., 2005). So far, eight different CRISPR-Cas systems subtypes have been identified, each subtype containing a marker *cas* gene along with a set of variable subtype-specific *cas* genes (Kunin et al., 2007).

Cyanophage infecting *M. aeruginosa* has been reported (Yoshida-Takashima et al., 2012; Ou et al., 2013), but few studies focus on the genomic features of defense system for this species. In our work, genomes of two strains of *M. aeruginosa* isolated from Taihu and Dianchi Lakes in China were sequenced and compared with the other available genomes of *M. aeruginosa* strains. The features of CRISPR array and diversity of CAS types in *M. aeruginosa* genomes were first elaborated here. We analyzed the potential function of the CRISPR-Cas system in resistance to exogenous genetic fragments by examining the spacers originated from phages, plasmids and environmental sequence data.

## Materials and methods

### Strain cultivation and DNA extraction

Two axenic strains of *M. aeruginosa*, TAIHU98 and DIANCHI905, were cultured in BG-11 medium at 25± 1°C under 25 μE m^−2^ s^−1^ with a 12/12 h light/dark cycle. Cells were harvested during the exponential growth phase then broken by Mini-Bead beater (Biospect Products, USA) at maximum speed. Genomic DNA was extracted via a modified method (Wu et al., 2000; Li et al., 2001) using SDS, proteinase K and lysozyme for cell-lysis, and phenol-chloroform-isoamylol for purification.

### Genome sequencing and annotation

The method and procedure for whole genome sequencing of TAIHU98 was described in Yang et al. (2013). For DIANCHI905, 300 bp paired-end and 3 kbp mate-paired libraries were constructed for Illumina/Solexa sequencing. *De novo* assembly was carried out by Velvet 1.08 (Zerbino and Birney, 2008) using only PE reads and then scaffolded by SSPACE (Boetzer et al., 2011) using both libraries. The Post Assembly Genome Improvement Toolkit (PAGIT) was used to generate a high quality draft genome by closing gaps, correcting sequence errors and transferring annotation (Swain et al., 2012). Putative ORFs were identified by Glimmer3 (Delcher et al., 2007) and Genemark (Besemer and Borodovsky, 2005). Protein functional annotations were determined by similarity searches against the NCBI nr, Pfam (Bateman et al., 2004) and COG (Tatusov et al., 2003) databases, and with Interproscan software (Quevillon et al., 2005). tRNA genes were predicted by tRNAscan-SE(Lowe and Eddy, 1997), while rRNA genes were identified by RNAmmer (Lagesen et al., 2007).

### Proteome comparison and definition of core- and pan-genomes

Genome data for *M. aeruginosa* strains was retrieved from NCBI. Each predicted proteome of the 14 strains was searched for orthologous genes against the total proteome by BLASTP (E ≥ 1e^−5^) analysis. Orthology between two proteins was defined as the best hit which had 50% identity over at least 50% of the length of both proteins. Then, all proteins were clustered into protein families using graph theory-based MCL (Markov Cluster algorithm). All these clusters together represented the size of the *M. aeruginosa* pan-genome, while clusters comprising genes shared by each genome are referred to as the core-genome. Core- and pan-genome plots of *M. aeruginosa* were drawn according to the method described in Reinhardt et al. (2009).

### BLASTP matrix and average nucleotide identity (ANI) calculation

A reciprocal BLASTP (e = 1e^−5^) for each pair of two proteomes was implemented according to 50/50 rule (Jacobsen et al., 2011; Ozen and Ussery, 2012) that is proteins must have at least 50% of total length shows 50% identity of the reference could be homology and assign to the same gene families. The two values in the matrix was the percentages for the shared gene families over the union genes in both query genomes, To reflect the whole genome relatedness on nucleotide level, ANI and tetranucleotide frequency correlation coefficients (TETRA) analysis were performed by JSpecies software (Richter and Rossello-Mora, 2009) based on MUMmer (Kurtz et al., 2004) algorithm implementation.

### Characterization of CRISPR-Cas systems

CRISPR were found using CRISPR-finder (http://crispr.u-psud.fr/Server/) with manual proofreading and the CRISPR comprised not less than three repeat units were considered as positive locus. The identification of *cas* genes was performed using BLAST against the Pfam and TIGRFAMs databases. Secondary structures of DR (direct repeat) sequences were predicted on the RNAfold web server (http://rna.tbi.univie.ac.at/cgi-bin/RNAfold.cgi). The search for similarities between each unique spacer was carried out by BLASTN (BLAST 2.2.29+) against a database limited to RefSeq databases of Plasmids, Bacteria and Viruses (date 2014-11-14), or the Env-nt Database (date 2014-11-14) at NCBI. The hits found within *M. aeruginosa* CRISPR loci were removed. Only hits with a bit score above 20 (corresponding to 100% identity over 20 bp) covering at least 25 bp were considered as proto-spacers (Pleckaityte et al., 2012). Sequence logos were generated on the Weblogo server (http://weblogo.berkeley.edu/) using 10 bp flanking sequences on both sides of the putative proto-spacers to search the proto-spacer adjacent motifs (PAM). The raw data of metagenome from Taihu lake was down load in NCBI Sequence Read Archive (Accession number SRA010762.3). *De novo* assembly used gsAssembler (Newbler, Roche) with default parameters and *Microcystis* sequences were picked up by BLASTN (E = 1e^−5^) using all the *M. aeruginosa* genomes as references. Then the identification of CRISPR repeats and spacers were carried out as abovementioned.

### Amplification and sequencing of CRISIPR arrays

Each CRISPR array in *M. aeruginosa* strain TAIHU98 and DIANCHI905 was amplified using a forward primer which specific to the leader region with a reverse primer which designed to be complementary to the 5′ end of the direct repeat with two additional nucleotides. This forward primer also amplified with a corresponding reverse primer which targeted the complementary to the 5′ end of unique spacer for sequencing the fragments. All primer sequences used in this study are shown in Table S1 in Supplementary Material. The PCR was performed with 25 μl containing 0.5 μl DNA (~50 ng), 0.5 μl each primer, 12.5 μl 2 × Taq PCR Master Mix (DBI Bioscience, German) and 11 μl H_2_O. The reaction conditions were as follows: 4 min of initial denaturing at 94°C, followed by 30 cycles of 94°C for 30 s and 57°C for 3 min with a final extension at 72°C for 10 min.

### Construction of phylogenetic trees

Unicellular cyanobacterial genomes containing at least one CRISPR locus and having a close relationship with *M. aeruginosa* according to the species tree in Cai et al. (2013) were downloaded from GenBank. *Cas1* genes from each genome were used to construct maximum-likelihood phylogenetic trees with PhyML 3.0. Thirty-one housekeeping genes (*dnaG, frr, infC, nusA, pgk, pyrG, rplA, rplB, rplC, rplD, rplE, rplF, rplK, rplL, rplM, rplN, rplP, rplS, rplT, rpmA, rpoB, rpsB, rpsC, rpsE, rpsI, rpsJ, rpsK, rpsM, rpsS, smpB*, and *tsf*) from representatives genomes were individually aligned using ClustalW (Thompson et al., 2002) and concatenated into a single alignment by Gblocks (Castresana, 2000). Species trees were inferred using the maximum likelihood method by RAxML v7.04 (bootstrap = 100) (Delsuc et al., 2005; Zhang et al., 2011). The radial tree was generated using iTOL (Letunic and Bork, 2007).

### Nucleotide sequence accession numbers

Whole Genome Shotgun sequencing projects of *M. aeruginosa* TAIHU98 and DIANCHI905 have been deposited at DDBJ/EMBL/GenBank with accession numbers ANKQ00000000 and AOCI00000000.

## Results

### Genome assembly and general features of *M. aeruginosa* genomes

Primary assembly of TAIHU98 used long reads by Newbler and resulted in 395 contigs, and paired-end reads were then used to assemble them into 50 contigs within 6 scaffolds. Gap-filling and error-correction were performed by the Phred-Phrap-Consed package. The finished TAIHU98 genome consists of only four contigs of 4,849,611 bp, with an average GC content of 42.45%. For DIANCHI905, trimmed paired-end reads were assembled into ~1000 contigs by Velvet, and then connected into ~700 contigs within 100 scaffolds by SSPACE using information from mate-pair reads. After an automatic finishing and merging process, the DIANCHI905 genome consists of 335 contigs (a total of 4,859,481 bp) with an N50 size of 27,753 bp and 42.45% GC content.

General genome information of *M. aeruginosa* strains used in comparative analysis is listed in Table 1. The genome size of *M. aeruginosa* is moderately flexible (4.23~5.84 Mb), but the GC content is very similar (around 42%). Although the number of proteins ranges from 4434 to 6312, the coding DNA sequence (CDS) density is ~81% in each genome. The genomes of *M. aeruginosa* strains NIES-843 and PCC 7806 were sequenced by traditional methods (Kaneko et al., 2007; Frangeul et al., 2008). Genomes for ten strains of *M. aeruginosa* were sequenced by NGS (Next Generation Sequencing) technology and their draft genomes were reported (Humbert et al., 2013). As shown in Table 1, the sequences of TAIHU98 and DIANCHI905 have fewer contigs and higher N50 sizes than the NGS-genomes from any other *M. aeruginosa* strains. Moreover, all 42 tRNA genes and two sets of rRNA clusters were completely identified in our genome sequences.

**Table 1:** **General information on *M. aeruginosa* strains used in comparative genome analysis**.

**Strain**	**Isolation Location**	**Year**	**Genome size(Mb)**	**GC%**	**Contigs No**.	**N50 length (bp)**	**Largest contig (bp)**	**CDS No**.	**tRNA No**.	**rRNA set**.	**Accession No**.
NIES-843	Lake kasumigaura, JP	1997	5.84	42.33	1	\	\	6312	42	2	AP009552.1
TAIHU98	Lake taihu, CN	1997	4.84	42.45	4	1,793,599	1,991,601	5356	42	2	ANKQ00000000.1
PCC 7806	Braakman Reservoir, NL	1972	5.19	42.43	116	91,379	533,374	5213	41	2	AM778843–AM 778958
DIANCHI905	Lake Dianchi, CN	1998	4.86	42.45	335	27,753	98,174	5571	42	2	AOCI00000000.1
PCC 9806	Oskosh, US	1975	4.26	43.1	310	26,394	108,279	4845	41	1	CAIL00000000.1
PCC 9432	Lake Lillte Rideau, CA	1954	4.99	42.54	438	24,301	87,129	4760	41	1	CAIH00000000.1
T1-4	Bangkok, TH	NA	4.69	42.78	449	23,234	73,081	4434	41	1	CAIP00000000.1
PCC 9808	Malpas dam, AU	1973	5.05	42.44	479	20,195	75,679	4845	41	1	CAIN00000000.1
PCC 7941	Lake Lillte Rideau, CA	1954	4.80	42.63	433	19,888	82,265	4520	41	1	CAIK00000000.1
PCC 9701	Guerlesquin dam, FR	1996	4.75	42.79	550	15,354	1,010,642	4483	41	1	CAIQ00000000.1
PCC 9443	Fish pond, Landijia, CF	1994	5.18	42.77	760	12,531	63,828	4780	41	1	CAIJ00000000.1
PCC 9807	Hartbeespoort dam, ZA	1973	5.15	42.66	782	11,789	85,874	4784	41	1	CAIM00000000.1
PCC 9809	Lake Michigan, US	1982	5.01	42.84	809	11,471	46,479	4680	41	1	CAIO00000000.1
PCC 9717	Rochereau dam, FR	1996	5.30	42.83	892	10,204	49,043	4836	41	1	CAII00000000.1

*Strains are listed in descending order of N50 size and CDS refers to coding sequences for proteins*.

### Core- and pan-genome reconstruction

**Figure 1:**
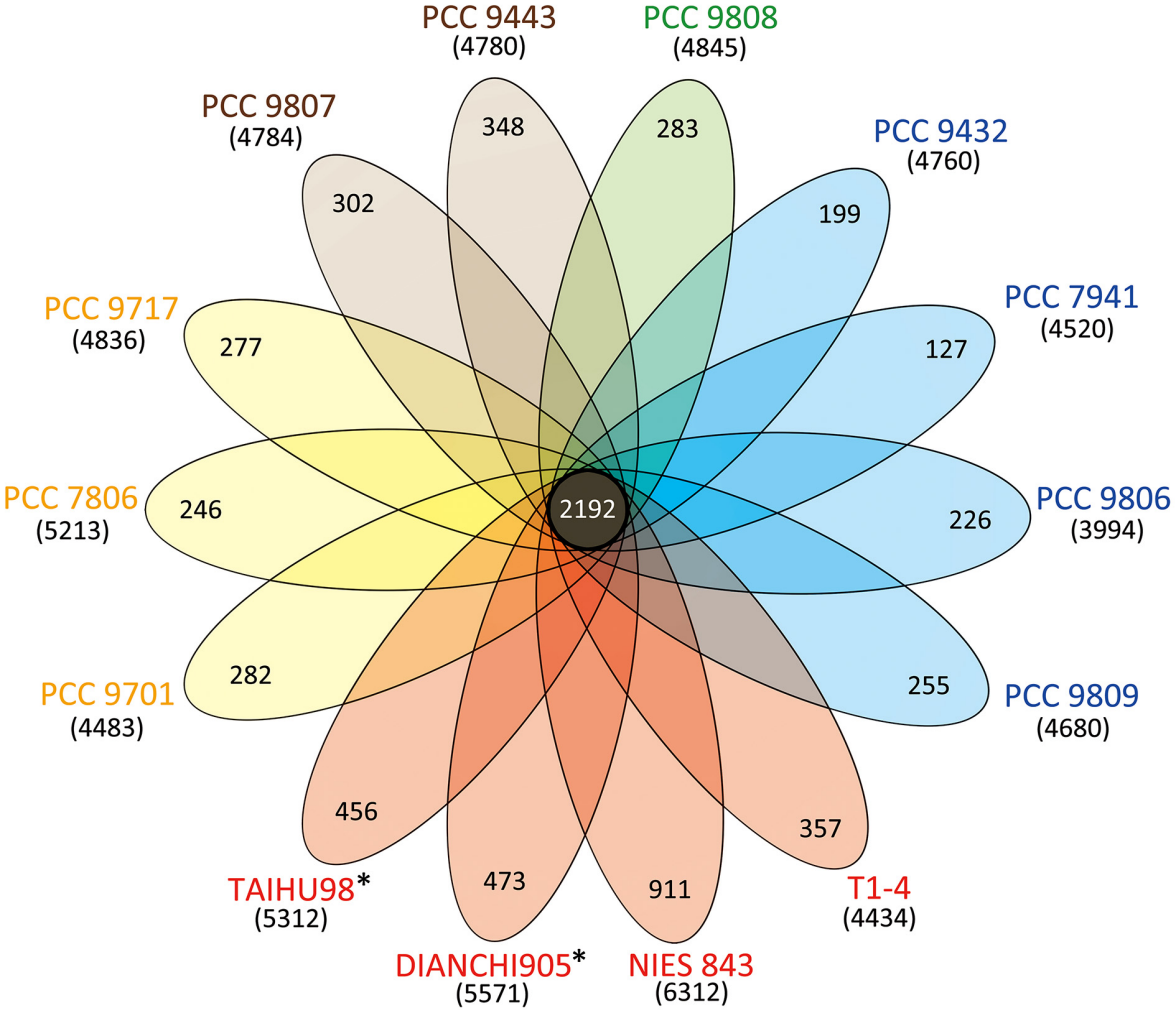
**Venn diagram displaying the genomic diversity of strains of *M. aeruginosa***. Each strain is represented by an oval that is colored according to the continent of isolation (Yellow, Europe; Red, Asia; Blue, America; Green, Oceania; Brown, Africa). The strains sequenced in this study are marked with^*^. The total number of protein coding genes within each genome is shown in brackets below the strain name. The size of the core-genome (orthologous coding sequences shared by all strains) is 2192. The number in the non-overlapping portions of each oval is the number of unique CDSs in each strain.

Figure 1 shows a high genomic diversity of *M. aeruginosa* strains. Its pan-genome comprises over 15,000 genes, roughly three times the average individual genome size. The highly conversed 2192 orthologous genes represent the core-genome for this species. The pan-genome is large and has not reached saturation. It will become larger as more strains are getting sequenced. Meanwhile the core-genome tends to be stable (Figure S1 in Supplementary Material). The core-genomic part accounted for 48.4 ± 4.6% in each genome, except for strain NIES-843 (34.7%) due to its unusual large genome size. Besides, each genome has many strain-specific genes, from 127 to 911, with correlation to the proteome size (rPearson = 0.84). Strain NIES-843 (14.4%), TAIHU98 (8.5%) and DIANCHI 905 (8.5%) have the three highest proportions of unique genes, significantly higher than the average level of 6.7%.

Total 4742 strain-specific genes for these genomes were detected and 77.7% of them were annotated as “hypothetical protein.” Although it is generally hard to assign them into a specific category in COG or GO database, 628 and 247 genes still show similarity to proteins from other organism or metagenome in the nr and Env-nr databases, respectively (Data Sheet 1 in Supplementary Material). These genes are assumed to be associated with traits to environmental adaptation, which might be obtained by horizontal gene transfer (HGT).

### BLASTP matrix and ANI analysis

BLASTP matrix and average nucleotide identity (ANI) value between two strains based on pair-wise genome comparisons were shown in Table 2. The BLASTp matrix percentage implicates the homology association within two strains on amino acid level. There is an average of 65.9% fraction for shared genes within the species, which indicates a considerable fraction of genes gained through-HGT. Pairs which took strain NIES-843 as reference genome have lower (=61.4%) fractions due to its largest proteome. Strain PCC 7806 and DIANCHI905 have the most share genes accounted 92.3% and 80.2% of its genome respectively. To determine if all these strains belong to *M. aeruginosa* species, ANI with TETRA analysis were performed as recommended. Two newly sequenced strains, together with another 12 genomes have an average of 96.02% ANI values with over 99.6% TETRA support. These values are higher than the ANI value of 94% suggested by Konstantinidis and Tiedje (2005). Pair-wise comparisons involved TAIHU98, PCC 9808, PCC 9432, and PCC 7941 even have ANI values constantly over 97.5%. The minimum ANI value (95.3%) is also above the threshold indicates that all these strains are belonging to same species and the species boundary of ANI value could be raise to 95%.

**Table 2:**
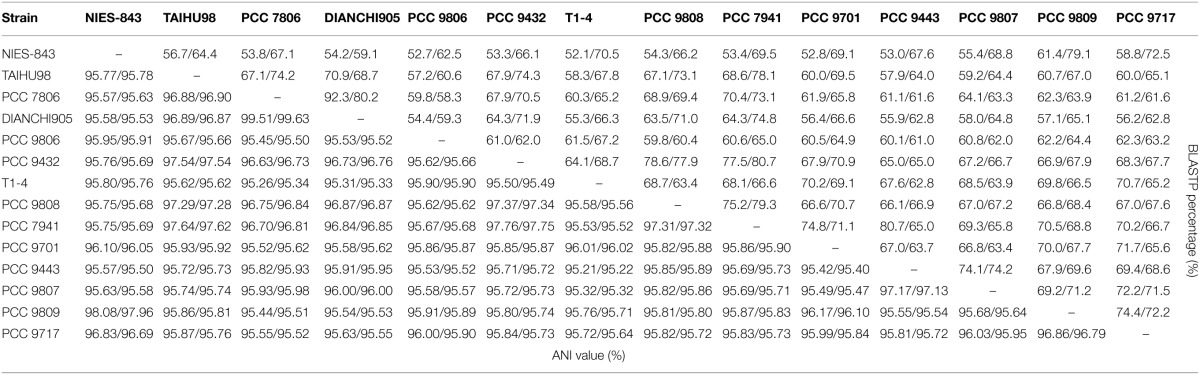
**BLASTP Matrix and ANI value show the homology within pairs of genome comparison on protein and nucleotide level**.

### Distribution of CRISPR arrays in *M. aeruginosa*

A total 71 of CRISPR loci containing seven types of DRs were identified in *M. aeruginosa* genomes (Table 3). The size of these CRISPR loci varied from ~0.26 kb (Cris-24) to ~13.6 kb (Cris-49), corresponding to from 3 to 187 repetitive units. Each strain has at least one CRISPR locus in its genome, suggesting a wide distribution of CRISPR among *M. aeruginosa*. This distribution is uneven. The strain PCC 9432 and PCC 9808 have abundant CRISPR arrays containing five types of DRs, the strains NIES-843, PCC 9806 and PCC 9809 have only one CRISPR locus, while the others have two to four CRISPR loci. CRISPR loci (Cris-4, 6, 8, 9, 13, 15, 16, 17) in TAIHU98 and DIANCHI905 were verified by CRISPR-based PCR and the electrophoretogram is shown in Figure S3 in Supplementary Material. The sequence of the PCR products confirmed CRISRP elements as we predicted from genome sequences.

**Table 3:** **General features of CRISPR loci in 14 *M. aeruginosa* genomes**.

**Strain name**	**CRISPR**	**Locus fragment**	**Direct Repe at sequence**	**DR type**	**Repeat No**.	**Spacer size (bp)**	**Start point**	**Length (kb)**	***cas* genes No**.	**CRISPR-Cas type**
NIES-843	Cris-1	NC_010296.1	GTTCCAATTAATCTTAAACCCTATTAGGGATTGAAAC	DR1	112	34–40	2814769	8.12	12	subtype I-D
	Cris-2	NC_010296.1	GTTCCAATTAATCTTAAACCCTATTAGGGATTGAAAC	DR1	20	34–40	2823103	1.49	–	
	Cris-3	NC_010296.1	GTTCCAATTAATCTTAAACCCTATTAGGGATTGAAAC	DR1	40	34–40	2826228	2.94	–	
TAIHU98	Cris-4	contig3	GTTCCAATTAATCTTAAACCCTATTAGGGATTGAAAC	DR1	45	33–43	584349	3.29	–	–
	Cris-5	contig3	GTTCCAATTAATCTTAAACCCTATTAGGGATTGAAAC	DR1	13	33–37	589278	0.97	–	–
	Cris-6	contig1	CTTGCTTCCAATTCGTGAAGCGTATGAATGGAAAC	DR2	15	35–41	306401	1.14	4	subtype III-B
	Cris-7	contig1	CTTGCTTCCAATTCGTGAAGCGTATGAATGGAAAC	DR2	18	33–41	310200	1.35	–	
	Cris-8	contig2	CTCTCTACTCGCTAGAGAAATTAATTGAATGGAAAC	DR3	13	35–41	1479390	0.99	4	subtype III-B
	Cris-9	contig2	GTTTCCAACTAATCCTATTTGACCTAATAGGTAAGG	DR4^a^	4	33–36	1341494	0.32	4	subtype III-B
PCC 7806	Cris-10	C326	GTTCCAATTAATCTTAAACCCTATTAGGGATTGAAAC	DR1	67	34–40	154383	4.91	8	subtype I-D
	Cris-11	C325	CTTGCTTCCAATTCGTGAAGCGTATGAATGGAAAC	DR2	10	35–39	179961	0.75	1	Incomplete
	Cris-12	C328	GTTTCCAACTAATCCTATTTGACCTAATAGGTAAGG	DR4^a^	13	34–41	195363	0.98	1	Incomplete
DIANCHI905	Cris-13	contig69	GTTTCAATCCCTAATAGGGTTTAAGATTAATTGGAAC	DR1^a^	67	34–40	4779	4.91	8	subtype I-D
	Cris-14	contig17	GTTTCCATTCATACGCTTCACGAATTGGAAGCAAG	DR2^a^	10	35–39	3432	0.75	1	Incomplete
	Cris-15	contig17	GTTTCCATTCATACGCTTCACGAATTGGAAGCAAG	DR2^a^	9	35–40	621	0.69	–	
	Cris-16	contig34	CTCTCTACTCGCTAGAGAAATTAATTGAATGGAAAC	DR3	78	35–41	13583	5.81	6	subtype III-B
	Cris-17	contig163	CCTTACCTATTAGGTCAAATAGGATTAGTTGGAAAC	DR4	13	34–41	1964	0.98	–	–
PCC 9806	Cris-18	AAI_E_2199_102	GTTTCCAACTAATCCTATTTGACCTAATAGGTAAGG	DR4^a^	13	34–42	5140	0.99	–	–
PCC 9432	Cris-19	AAI_A_2195_289	GTTTCAATCCCTAGTAGGGTTTAAGATTAATTGGAAC	DR1^a b^	33	34–38	3124	2.41	5	subtype I-D
	Cris-20	AAI_A_2195_207	GTTTCCATTCATACGCTTCACGAATTGGAAGCAAG	DR2^a^	12	34–44	58697	0.92	4	subtype III-B
	Cris-21	AAI_A_2195_207	GTTTCCATTCATACGCTTCACGAATTGGAAGCAAG	DR2^a^	9	33–43	61994	0.69	–	
	Cris-22	AAI_A_2195_73	GTTTCCATTCAATTAATTTCTCTAGCGAGTAGAGAG	DR3^a^	51	34–39	16722	3.75	6	subtype III-B
	Cris-23	AAI_A_2195_162	GTTTCCAACTAATCCTATTTGACCTAATAGGTAAGG	DR4^a^	5	37–38	8964	0.40	4	subtype III-B
	Cris-24	AAI_A_2195_163	CCTTACCTATTAGGTCAAATAGGATTAGTTGGAAAC	DR4	3	39–44	2709	0.26		
	Cris-25	AAI_A_2195_2	GTGATCAACGCCTTACGGCATCAAAGGTTAGTACAC	DR7	17	34–38	746	1.25	7	subtype I-A
T1-4	Cris-26	AAI_I_2203_92	CTTACCTATTAGGTCAAATAGGATTAGTTGGAAAC	DR4^c^	13	34–40	409	0.99	–	–
	Cris-27	AAI_I_2203_144	GTTTCCATTAATTAAACTTGCTAAGAAGTTAAAAG	DR5^a^	18	33–49	87	1.36	–	–
PCC 9808	Cris-28	AAI_G_2201_117	GTTCCAATTAATCTTAAACCCTACTAGGGATTGAAAC	DR1^b^	87	34–42	15318	6.37	8	subtype I-D
	Cris-29	AAI_G_2201_119	GTTCCAATTAATCTTAAACCCTACTAGGGATTGAAAC	DR1^b^	15	34–42	21	1.13	–	
	Cris-30	AAI_G_2201_119	GTTCCAATTAATCTTAAACCCTACTAGGGATTGAAAC	DR1^b^	18	33–40	1313	1.33	–	
	Cris-31	AAI_G_2201_276	CTTGCTTCCAATTCGTGAAGCGTATGAATGGAAAC	DR2	11	36–42	232	0.85	4	subtype III-B
	Cris-32	AAI_G_2201_275	CTTGCTTCCAATTCGTGAAGCGTATGAATGGAAAC	DR2	14	33–43	47750	1.06	–	
	Cris-33	AAI_G_2201_381	CCTTACCTATTAGGTCAAATAGGATTAGTTGGAAAC	DR4	14	33–42	416	1.07	–	–
	Cris-34	AAI_G_2201_250	GTGATCAACGCCTTACGGCATCAAAGGTTAGTACAC	DR7	56	33–38	2189	4.04	6	subtype I-A
	Cris-35	AAI_G_2201_359	CTTTCATCTCTTACTCCCCGCAAGGGGACGGAAAC	DR6	10	36–49	2466	0.77	6	subtype III-A
	Cris-36	AAI_G_2201_361	CTTTCATCTCTTACTCCCCGCAAGGGGACGGAAAC	DR6	7	34–36	5211	0.52		
PCC 7941	Cris-37	AAI_D_2198_350	GTTCCAATTAATCTTAAACCCTATTAGGGATTGAAAC	DR1	43	33–42	2965	3.14	8	subtype I-D
	Cris-38	AAI_D_2198_351	GTTCCAATTAATCTTAAACCCTATTAGGGATTGAAAC	DR1	35	34–44	36	2.60	–	
	Cris-39	AAI_D_2198_123	GTTTCCATTCATACGCTTCACGAATTGGAAGCAAG	DR2^a^	11	36–42	3718	0.85	1	Incomplete
	Cris-40	AAI_D_2198_123	GTTTCCATTCATACGCTTCACGAATTGGAAGCAAG	DR2^a^	10	33–41	6693	0.76	–	
	Cris-41	AAI_D_2198_26	CCCTCTACTCGCTAGAGAAATTAATTGAATGGAAAC	DR3^b^	22	35–42	13597	1.65	6	subtype III-B
	Cris-42	AAI_D_2198_372	GTTTCCAACTAATCCTATTTGACCTAATAGGTAAGG	DR4^a^	8	34–44	6371	0.64	4	subtype III-B
PCC 9701	Cris-43	AAI_K_2204_125	GTTTCAATCCCTAATAGGGTTTAAGATTAATTGGAAC	DR1^a^	66	33–40	37	4.76	7	subtype I-D
	Cris-44	AAI_K_2204_1	GTTCCAATTAATCTTAAACCCTATTAGGGATTGAAAC	DR1	20	34–39	34	1.47	–	
	Cris-45	AAI_K_2204_scaffold94	GTTTCAATCCCTAGTAGGGTTTAAGATTAATTGGAAC	DR1^ab^	19	34–40	42	1.42	–	
	Cris-46	AAI_K_2204_scaffold117	GTTCCAATTAATCTTAAACCCTATTAGGGATTGAAAC	DR1	9	34–36	35	0.69	–	
	Cris-47	AAI_K_2204_scaffold147	GTTCCAATTAATCTTAAACCCTATTAGGGATTGAAAC	DR1	12	33–41	27	0.90	–	
	Cris-48	AAI_K_2204_22	CTCTCTACTCGCTAGAGAAATTAATTGAATGGAAAC	DR3	26	35–42	11353	1.95	6	subtype III-B
PCC 9443	Cris-49	AAI_C_2197_158	GTTCCAATTAATCTTAAACCCTATTAGGGATTGAAAC	DR1	187	34–40	23210	13.61	12	subtype I-D
	Cris-50	AAI_C_2197_158	GTTCCAATTAATCTTAAACCCTATTAGGGATTGAAAC	DR1	22	32–39	36952	1.62	–	
	Cris-51	AAI_C_2197_217	GTTCCAATTAATCTTAAACCCTATTAGGGATTGAAAC	DR1	42	31–40	3	3.07	–	
	Cris-52	AAI_C_2197_246	GTTTCCAACTAATCCTATTTGACCTAATAGGTAAGG	DR4^a^	11	35–46	16661	0.87	4	subtype III-B
	Cris-53	AAI_C_2197_246	CCTTACCTATTAGGTCAAATAGGATTAGTTGGAAAC	DR4	6	37–42	25855	0.49		
	Cris-54	AAI_C_2197_143	CTTTTAACTTCTTAGCAAGTTTAATTAATGGAAAC	DR5	21	34–50	633	1.60	–	–
PCC 9807	Cris-55	AAI_F_2200_415	GTTTCAATCCCTAATAGGGTTTAAGATTAATTGGAAC	DR1^a^	83	34–41	496	6.08	8	subtype I-D
	Cris-56	AAI_F_2200_191	CTTGCTTCCAATTCGTGAAGCGTATGAATGGAAAC	DR2	11	29–42	12084	0.83	4	subtype III-B
	Cris-57	AAI_F_2200_191	CTTGCTTCCAATTCGTGAAGCGTATGAATGGAAAC	DR2	17	34–45	15137	1.29	–	
	Cris-58	AAI_F_2200_74	CCTTACCTATTAGGTCAAATAGGATTAGTTGGAAAC	DR4	6	37–42	3528	0.49	2	subtype III-B
	Cris-59	AAI_F_2200_510	CTTTTAACTTCTTAGCAAGTTTAATTAATGGAAAC	DR5	13	33–44	3271	1.01	–	–
	Cris-60	AAI_F_2200_508	CTTTTAACTTCTTAGCAAGTTTAATTAATGGAAAC	DR5	8	33–45	4075	0.62	–	–
PCC 9809	Cris-61	AAI_H_2202_401	GTTCCAATTAATCTTAAACCCTATTAGGGATTGAAAC	DR1	35	34–39	1310	2.57	8	subtype I-D
PCC 9717	Cris-62	AAI_B_2196_565	GTTCCAATTAATCTTAAACCCTATTAGGGATTGAAAC	DR1	83	34–41	3668	6.06	8	subtype I-D
	Cris-63	AAI_B_2196_566	GTTCCAATTAATCTTAAACCCTACTAGGGATTGAAAC	DR1^b^	25	33–43	35	1.84	–	
	Cris-64	AAI_B_2196_854	GTTCCAATTAATCTTAAACCCTACTAGGGATTGAAAC	DR1^b^	25	33–42	60	1.84	–	
	Cris-65	AAI_B_2196_798	GTTTCAATCCCTAATAGGGTTTAAGATTAATTGGAAC	DR1^a^	11	33–37	38	0.83	–	
	Cris-66	AAI_B_2196_800	GTTCCAATTAATCTTAAACCCTATTAGGGATTGAAAC	DR1	6	34–40	35	0.47	–	
	Cris-67	AAI_B_2196_826	GTTCCAATTAATCTTAAACCCTATTAGGGATTGAAAC	DR1	14	34–36	73	1.04	–	
	Cris-68	AAI_B_2196_831	GTTCCAATTAATCTTAAACCCTATTAGGGATTGAAAC	DR1	11	34–45	11	0.84	–	
	Cris-69	AAI_B_2196_87	CCTTACCTATTAGGTCAAATAGGATTAGTTGGAAAC	DR4	18	35–42	5103	1.37	4	subtype III-B
	Cris-70	AAI_B_2196_91	CTTTTAACTTCTTAGCAAGTTTAATTAATGGAAAC	DR5	14	34–42	2646	1.06	–	–
	Cris-71	AAI_B_2196_92	CTTTTAACTTCTTAGCAAGTTTAATTAATGGAAAC	DR5	8	33–42	48	0.62	2	Incomplete

*DR type^a^ is reverse compliment strand; DR type^b^ refers to the mutation of “T” to “C” at the 25th base; DR type^c^ indicates the deletion of “C” at the first base. Cas type was defined in (Makarova et al., 2011)*.

Some CRISPR loci with the same DR are located next to each other (e.g., Cris-20~21) or on adjacent contigs (e.g., Cris-31~32), thus could be seen as a bigger, entire CRISPR arrays in genome. These interruptions are possibly due to poor assembly caused by long repetitive sequence such as IS elements on sides of CRISPR array as reported (Horvath et al., 2009; Kuno et al., 2012). The majority of CRISPR arrays have *cas* genes nearby. More than one type of *cas* gene was identified in other 12 strains, whereas no *cas* gene was identified in T1-4 or PCC 9806, indicating incomplete CRISPR-Cas system for these two strains.

### Architecture of CRISPR-Cas systems

**Figure 2:**
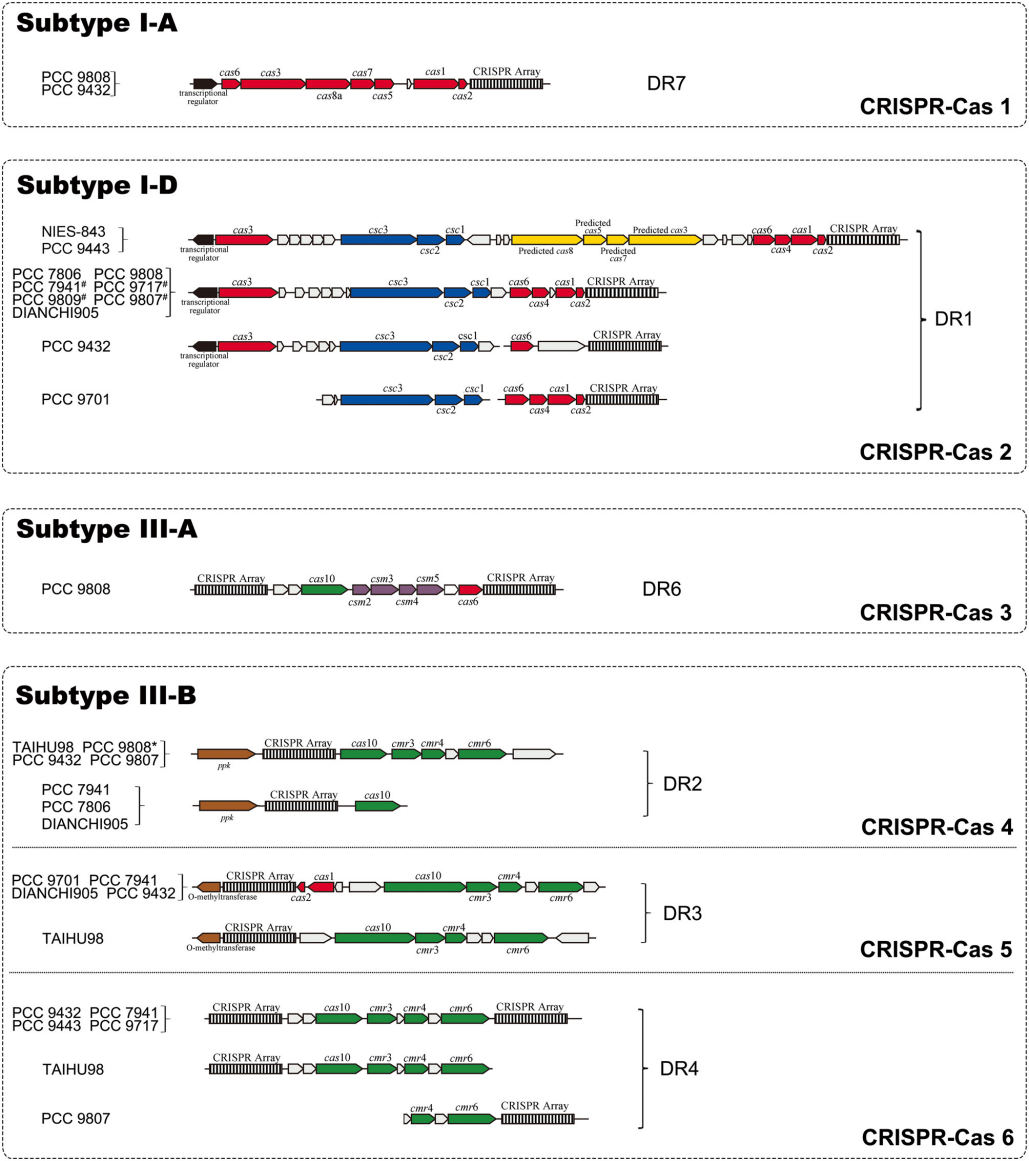
**Organization of four CRISPR-Cas systems identified in *M. aeruginosa* strains**. CRISPR arrays are depicted as black-and-white boxes. Arrows in red and yellow represent genes named as “*cas*” and “predicted *cas*,” respectively. Arrows in blue, green and purple illustrate “*csc*,” “*cmr*” and “*csm*” genes, respectively. Empty arrows refer to conserved hypothetical proteins while black arrows display the transcriptional regulator located upstream of CRISPR-Cas 1 and 2. The known genes coding for polyphosphate kinase (*ppk*) and O-methyltransferase located downstream of CRISPR-Cas 3 and 4 are represented by brown arrows. Genes for subtype I-D separated in adjacent fragments in strains which marked by “^#^.” PCC 9808 with a superscript “^*^” means no *ppk* gene was found.

We searched upstream and downstream of each CRISPR locus for genes encoding CRISPR-associated proteins (CAS) and found four types of *cas* gene cluster. In addition to the previously reported subtype I-D *cas* gene sets, three CAS subtypes I-A, III-A and III-B were identified and described here (Figure 2). Seven genes (*cas6, cas*3, *cas8a, cas7, cas5, cas1, cas2*) constituted the subtype I-A CRISPR-Cas 1, which was only found in strains PCC 9808 and PCC 9432. Subtype I-D CRISPR-Cas 2, which consisted of eight genes (*cas3, csc3/cas10d, csc2, csc1, cas6, cas4, cas1, cas2*), was found in 12 of the 14 sequenced *M. aeruginosa* strains. Four additional predicted genes (predicted *cas8a*, predicted *cas5*, predicted *cas7*, predicted *cas3-I*) as defined by Kuno et al. (2012) in strains NIES-843 and PCC 9443 and they had no significant hits when searched against TIGRFAMs. CRISPR-Cas 1 has an upstream transcriptional regulator. So does CRISPR-Cas 2, but in the opposite direction of transcription of the *cas* cluster, suggesting transcriptional regulator is essential for the function of type I CRISPR-Cas system. Only strain PCC 9808 had CRISPR-Cas 3, which was identified as subtype III-A, and contained six genes (*cas10/cmr2, csm3, csm4, csm5, csm6, cas6*). Subtype III-B CRISPR-Cas 4 and 5 contains four main genes (*cas10/cmr2, cmr3, cmr4, cmr6*) and a *ppk* or O-methyltransferase coding gene in upstream. CRISPR-Cas 6 also contains the above four *cas* genes but with a CRISPR array upstream and downstream. The sets of *cas* genes of the same type in different strains are highly conserved even where there are proteins with unknown functions in between. The *cas3* genes in subtypes I-A and I-D are not identical proteins but are within the same family, so does *cas6*. On inspecting the organization of the *cas* genes of CRISPR-Cas 2 and CRISPR-Cas 5, it was noted that *cas1* and *cas2* were missing in PCC 9432 and TAIHU98. This was verified by PCR in TAIHU98 to exclude assembly errors.

### Evolutionary relationships inferred by Cas1 protein tree

**Figure 3:**
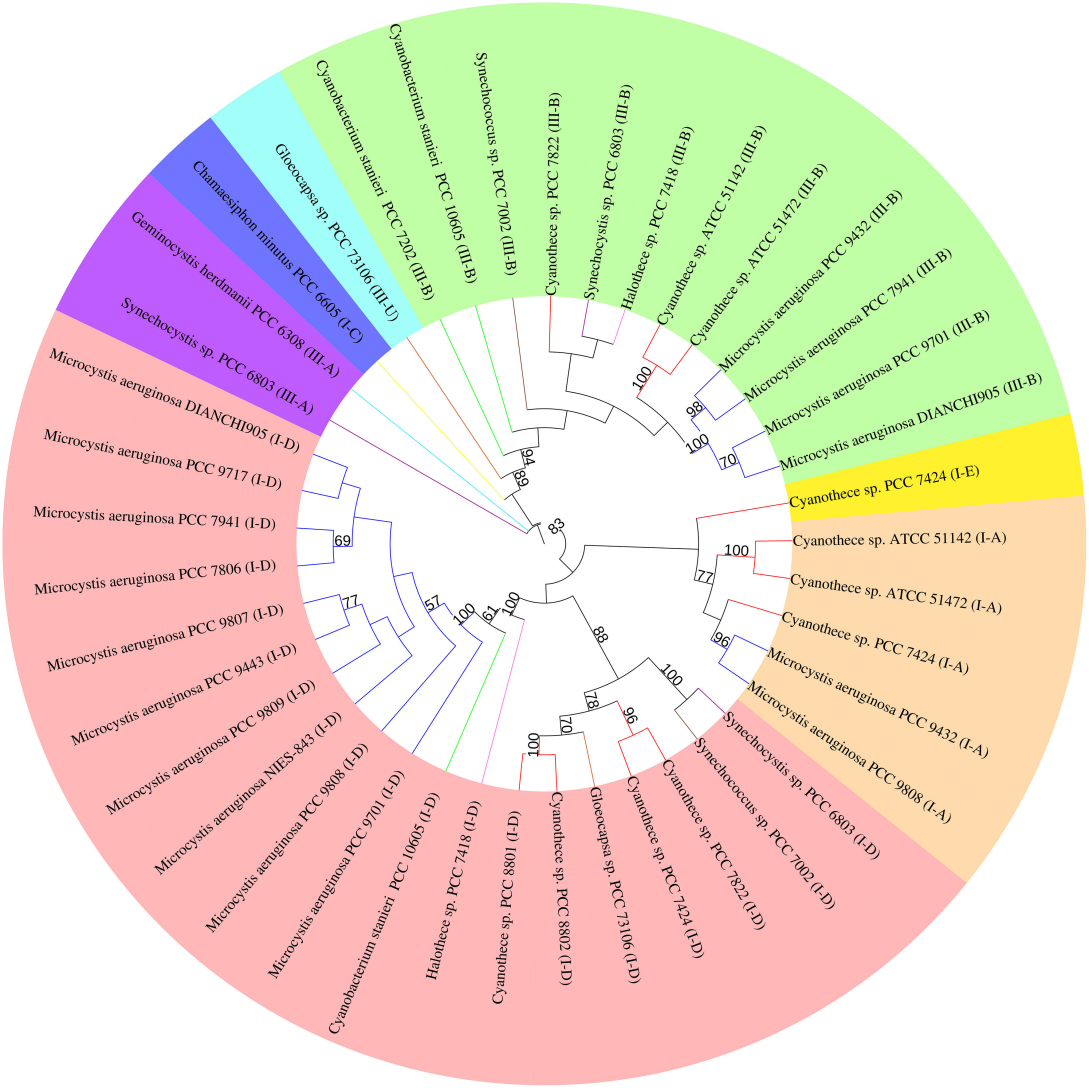
**Phylogenetic trees of Cas1 proteins of *M. aeruginosa*and closely related cyanobacterium**. Phylogenetic tree based on the Cas1 proteins from different CRISPR-Cas system types in each genome.*cas1* genes belonging to different CRISPR-Cas systems are clustered into groups and highlighted by color. Branches are colored according to species (Dark blue, *M. aeruginosa*; Red, *Cyanothece* spp.; Purple, *Synechocystis* spp.; Brown, *Syechococcus* spp.; Yellow, *Chamaesiphon minutus*; Orange, *Gloeocapsa* spp.; Pink, *Halothece* spp.; Green, *Cyanobacterium stanieri*; Light blue, *Geminocystis herdmanii*). Only branches with bootstrap values over 50 (out of 100 replicates) are shown. The colors in the outer ring refers to the origin type of Cas1 (Pink, I-D; Orange, I-A; Dark blue, I-C; Yellow, I-E; Green, III-B; Sky blue, III-U; Purple, III-A).

The highly conserved Cas1 protein can be used as a marker to investigate the evolution of the CRISPR-Cas system. The other universal protein, Cas2, is too small to yield a well resolved tree. Forty-one Cas1 protein sequences from 14 *M. aeruginosa* and 14 closely related cyanobacteria were used in our study (Data Sheet 2 in Supplementary Material). The phylogenetic tree (Figure 3) includes several well-resolved branches that generally agree with the clear classification of CRISPR-Cas systems into subtypes I-A, I-D, III-A, and III-B, with the few notable exceptions of subtypes I-E, I-C, and III-U. The majority of strains have subtype I-D (19/28 strains) and subtype III-B (12/28 strains) Cas1 protein, indicating that these two subtype systems might be conservative in the freshwater unicellular cyanobacteria. Subtype I-D separates into two subclades, in which proteins from same species group together. All Cas1 assigned to subtype I-A cluster together with a single subtype I-E Cas1 of *Cyanothece* sp. PCC 7424. All subtype III-B Cas1 proteins cluster together, along with the only subtype III-U Cas1 protein from *Gloecocapsa* sp. PCC 73106. The Cas1 phylogeny has poor congruence with the major subclades of a species tree based on 31 housekeeping genes (Figure S2 in Supplementary Material). *M. aeruginosa* displays much closer relationships with *Cyanothece* spp. PCC 7424 and PCC 7822 than with *Cyanobacterium stanieri* PCC 10605 or *Halothece* sp. PCC 7418 in the species tree, but the opposite result was obtained when considering the Cas1 of subtype I-D from those organisms. The 31 housekeeping genes are highly conserved in bacteria and involved in information processing (replication, transcription and translation) or central metabolism, and thus are thought to be relatively recalcitrant to horizontal gene transfer (Jain et al., 1999). The species tree overcame the defect of phylogenetic analysis using a single protein sequence by analyzing longer, multiple proteins and thus provided powerful evidence of the evolutionary relationships of the core, “calm” part of the genomes. The inconsistency between these two trees suggests that Cas1 has undergone a different evolutionary process than the core genes. This leads to a possible explanation that the CRISPR-Cas system has a high propensity for horizontal gene transfer (Godde and Bickerton, 2006; Tyson and Banfield, 2008).

### Characterization of CRISPR repeat families

**Figure 4:**
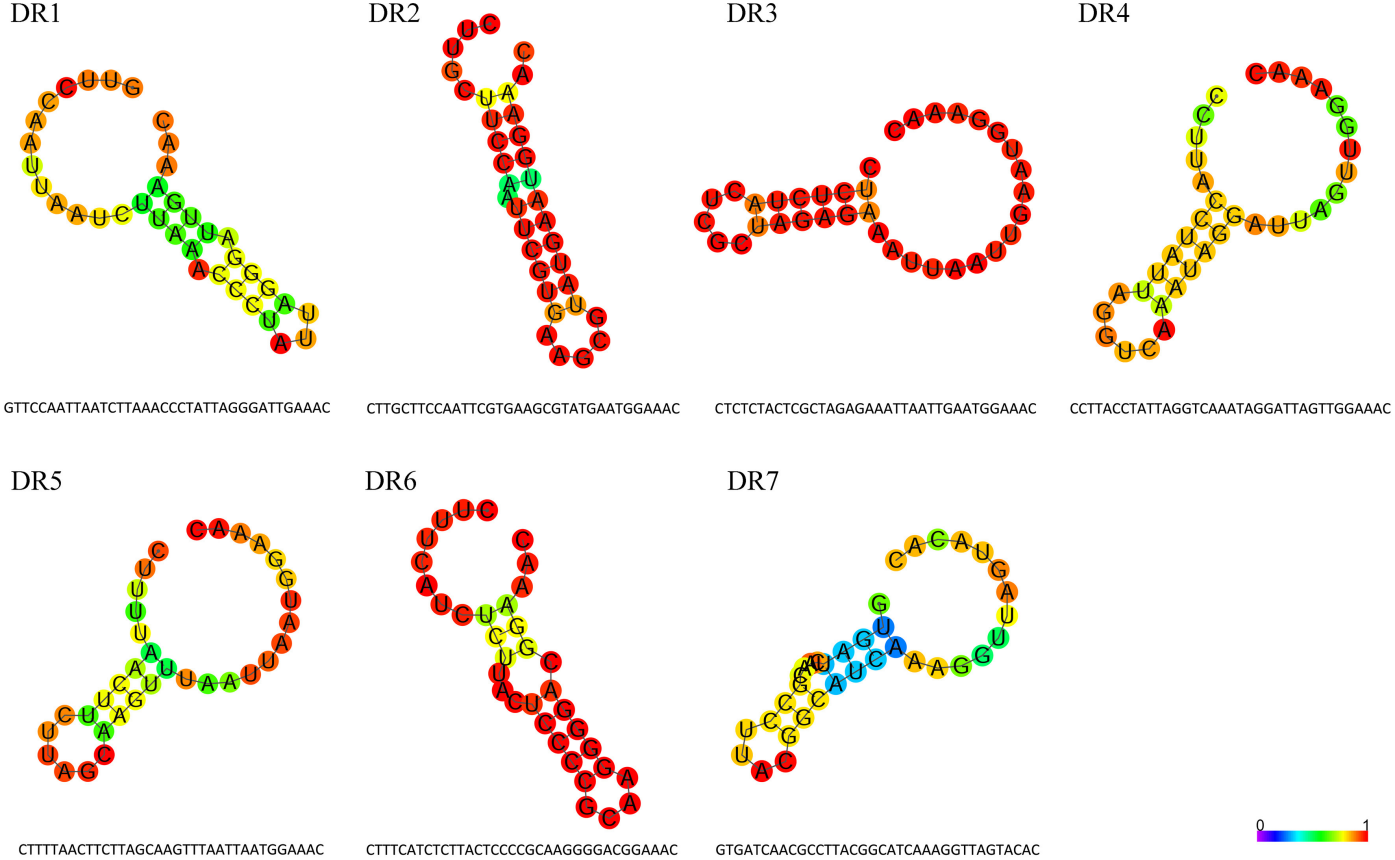
**Features of direct repeat sequences in *M. aeruginosa* CRISPR arrays**. Sequences and predicted secondary structures of the seven types of direct repeat in *M. aeruginosa*CRISPR arrays. The structures are colored by base-paring probabilities; for unpaired regions colors denote the probability of being unpaired.

CRISPRs are typically defined by their repeat sequence. DR sequences of all CRISPRs in *M. aeruginosa* genomes were clustered into seven types with length ranges from 35 to 37 nt. These DRs are partially palindromic and form putative RNA secondary structures as shown in Figure 4. The stem-loop in all DR highlights its importance for the functionality of CRISPR-Cas systems. All DRs except DR7 have a conserved 3′ terminus of GAAAC, possibly acting as a binding site for Cas proteins according to previous reports (Kunin et al., 2007). The repeat DR7 has a TACAC 3′ terminal sequence, which might indicate a specific signature for this particular set of CRISPR repeat families. DR6 and DR1 have quite similar secondary structures and both belong to the same CRISPR-Cas system, suggesting the type I CRISPR-Cas system might rely on such a secondary structure. Some repeats may be slightly different from the others because of single nucleotide polymorphisms (Horvath et al., 2009), for example, the mutation of A/T to G/C in 24th base within DR1 has no effect on the structure, so we consider them as the same DR type. Besides, DR1 is the repeat mostly distributed and is normally associated with the longest CRISPR array.

A close association between CRISPR DR and CAS types was observed clearly (Table S2 in Supplementary Material). CRISPR loci containing DR1 exclusively appear downstream of the subtype I-D *cas* cluster, while DR6 is closely tied in with subtype I-A. Similarly, DR7 corresponds strictly to subtype III-A. CRISPR loci of DR2, DR3, and DR4 invariably locate with subtype III-B *cas* gene sets. We found no *cas* genes when searching flanking sequences of CRISPR loci comprising DR5. Possible reasons for this could be an insufficient number of sequenced strains and/or poor genome assembly. The overall pronounced correspondence between the CAS subtypes and DR types in *M. aeruginosa* species has also been noticed in other species but was somewhat flexible (Kunin et al., 2007). This association confirmed that different and specific sets of *cas* genes are important to recognize, bind and process the different repeat types, especially for a specific species such as *M. aeruginosa*.

### Analysis of CRISPR spacer sequences

**Figure 5:**
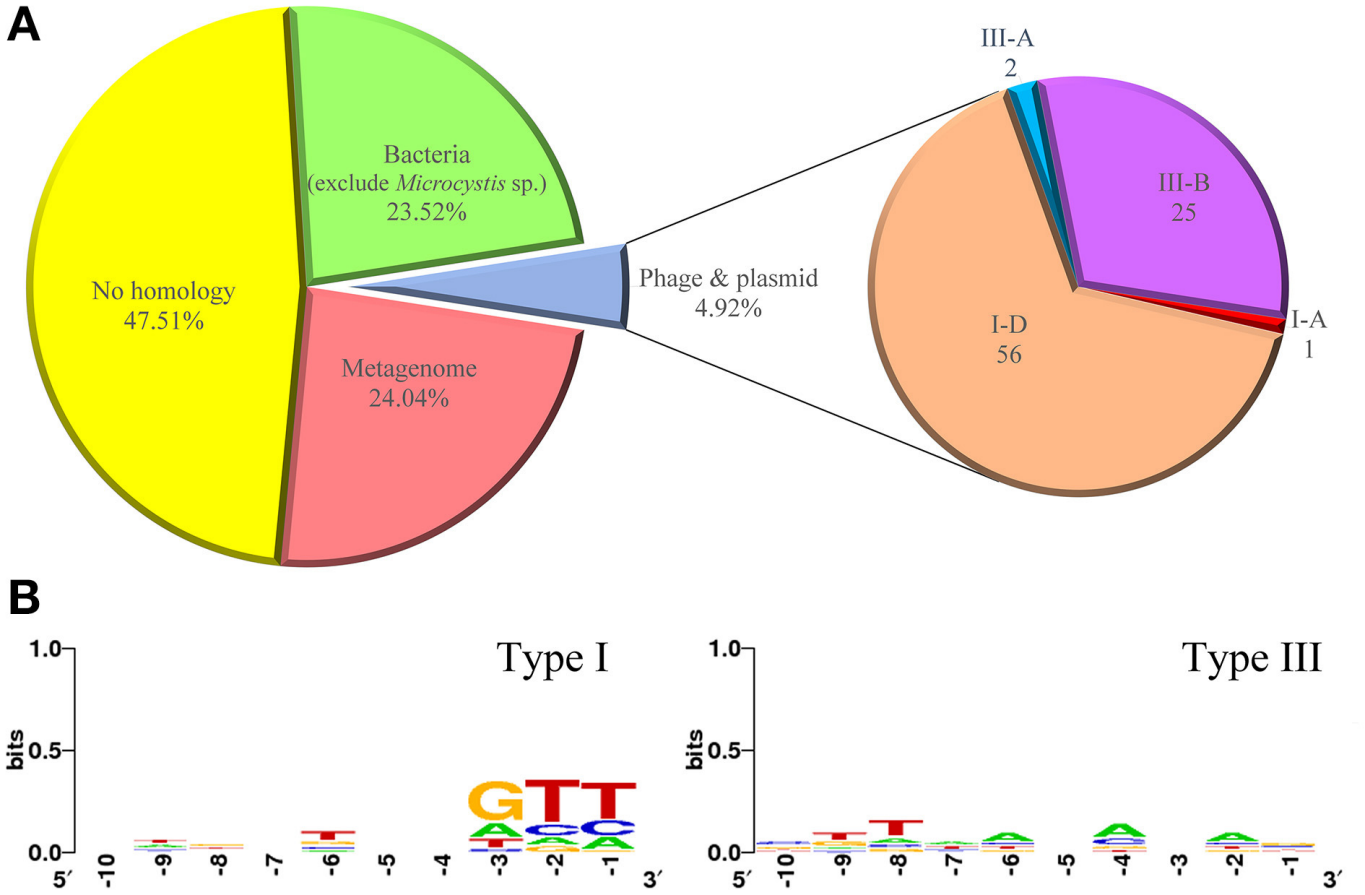
**Percentage of spacer matches to exogenous DNA fragments and putative PAM consensus of protospacers. (A)** Matches of CRISPR spacers identified in *M. aeruginosa* strains to metagenomes, plasmids, phage, and chromosomal bacteria. **(B)** Sequence logo of PAM consensus for type I CRISPR-Cas system and non PAM consensus for type III CRISPR-Cas system. Numbers below the sequence data indicate nucleotide positions where −1 is just upstream of the proto-spacers.

The spacer repertoire at each locus represents a history of previous invasions. We identified a total of 1726 unique spacers (ranging from 29 to 54 nt) in all CRISPR loci of the *M. aeruginosa* genomes and compared them with available databases to search for possible proto-spacers from bacteria, plasmids, viruses or metagenome data (Figure 5A). After excluding hits from *M. aeruginosa* itself, 404 spacers (23.52%) showed similarity to chromosomal sequences of bacteria, including some cyanobacterial strains. Only 84 spacers (4.92%) had significant similarities (90~100%) to known plasmids and phages which could be used to infer their putative proto-spacer adjacent motifs (PAM). However, there were 806 spacers (47.51%) showing no homology to any organism or sequencing data in database. Such low correspondence between CRISPR spacers and extra-chromosomal elements is consistent with previous studies (Deveau et al., 2008; Horvath et al., 2008), and reflects the bias in the data available in public databases.

Microorganisms with sequenced genomes in databases today represent only a very small portion of these diverse and abundant organisms. Traditional microbial genome sequencing and genomics based on cultivated cultures could not fully reveal the vast majority of microbial biodiversity, because over 99% bacteria present in natural environments cannot be cultured (Hugenholtz et al., 1998; Rappe and Giovannoni, 2003). We therefore introduced the Env-nt database, which contains DNA sequenced directly from the environment, to provide links between the spacer signatures and uncultured microorganism, and thus reduce the percentage of unknown hits. We found that 414 spacers (24.04%) matched to the environmental sequencing data (mainly from marine or freshwater metagenomes), suggesting CRISPR-Cas systems in *M. aeruginosa* may target a wide range of exogenous DNA fragments in the environment. We further analyzed CRISPR features of metagenome data from Taihu Lake (see Materials and Methods) and found that 26 CRISPR arrays on the contigs associated to *Microcystis*, Four and Only four types of DR, which was the same as identified in *M. aeruginosa* TAIHU98, were detected. Ten spacers from the metagenome data show similarity to the non-*Microcystis* sequences (Data Sheet 4 in Supplementary Material).

Of the 84 spacers matched to known plasmids and phages (Data Sheet 3 in Supplementary Material), 56 spacers were from subtype I-D CRISPRs, 25 from subtype III-B CRISPRs. Only one and two spacers were from subtypes I-A and III-A CRISPRs, respectively. Eighteen spacers across nine strains show identity to phage Ma-LMM01 (AB231700). Fourteen, four and eight unique spacers matching to *M. aeruginosa* associated plasmids pMA1 (NC_002060.1), pMA2 (NC_001597.1) and PMA1 (Z28337.1), respectively, were also found in strains PCC 9807, PCC 9808, PCC 9701, PCC 9717, NIES-843, and TAIHU98. Besides, 29 spacers show similarity to plasmids from other bacteria and 11 spacers show similarity to phages. No spacers in strains T1-4 or PCC 9806 were identified as originating from plasmid or phage. As mentioned above, we suggest these two strains without complete CRISPR-Cas system lack the activity for resistance to exogenous DNA, so no trace of phage or plasmid was left.

In CRISPR defending systems, where an invading nucleic acid is excised into proto-spacers is not random, but depends on short (3~5 bp) DNA sequences called proto-spacer adjacent motifs (PAM). The PAM sequence varies with CRISPR-Cas type (Mojica et al., 2009). In type I and II systems, PAMs are essential for target recognition, and a short seed sequence within the match is required in particular subtypes. For type III systems, no PAMs have been identified so far and it is unclear whether a seed sequence exists (Deveau et al., 2008; Marraffini and Sontheimer, 2008; Cady et al., 2012). Figure 5B shows the putative PAMs for CRISPRs identified in *M. aeruginosa*. *M. aeruginosa* spp. contain four subtypes of CRISPR-Cas belonging to type I and type III systems, and some strains even have more than two types of CRISPR-Cas system within their genome. Considering this, sequences for PAM searching were divided into two groups. No obvious motif was detected when analyzing downstream sequences for both systems. But a weak GTT motif in close proximity to upstream was identified for type I CRISPR-Cas. The ambiguous positions of PAM for type I system could be explained by the diversity of the spacer donors. For type III system, none explicit base was observed upstream from the proto-spacers sequences.

## Discussion

Whole genome sequencing of TAIHU98 and DIANCHI905 from two China lakes enriches the species genome records of Asia. Collectively, sequenced *M. aeruginosa* strains have been covered all the five continents of the Earth. The high-quality assembly of *M. aeruginosa* genomes is the key point to explore genome features and genomic comparison analysis. Many strain-specific genes were found in each *M. aeruginosa* genome, indicating a choppy, open pan-genome for *M. aeruginosa*, as reported for *Streptococcus* spp. (Lefebure and Stanhope, 2007; Donati et al., 2010) and *Escherichia coli* (Lukjancenko et al., 2010). These unique genes are assumed to beneficial for survival and proliferation of the species and probably gained through horizontal gene transfer (HGT). Besides, large proportion of repeat sequences and low synteny values between strains of which involved genome rearrangement were revealed by previous research (Humbert et al., 2013). Above all, *M. aeruginosa* genomes display highly plasticity on both genomic contents and genomic organization.

However, the genomic plasticity of *M. aeruginosa* is not unlimited, *M. aeruginosa* maintains the highly conserved core-genome thus keeps species identity authentication according to the result of ANI analysis. Before genome age, classical DNA-DNA hybridization (DDH) technique with characteristic phenotypic traits is the preferred for genetically determining bacterial species. The empirical evidences based on classified species and their comparisons with DDH values lead to the recommendation that over 70% re-association could be a criterion in this category circumscription (Goris et al., 2007). Recently, the ANI analysis is recommended to substitute DDH for bacterial species circumscription (Richter and Rossello-Mora, 2009). Each pair of ANI value is above the threshold (94%) indicates that all these strains are still belonging to same species. On the other hand, for confining the plasticity, effective restriction of HGT by defense mechanisms, especially the CRISPR-Cas systems, are essential for genome stability.

As a free-living cyanobacterium spreading a wide range of freshwater ecosystems, *M. aeruginosa* is frequently exposed to many kinds of stresses such as invasion by bacteriophages or conjugation of plasmids. To survive this invasion or to restrict the transfer of exogenous DNA, sophisticated defense mechanisms against foreign DNA was evolved in *M. aeruginosa*. In cyanobacteria, defense mechanisms posing various physical barriers (natural competence and the exopolysaccharide layer) and biochemical (restriction-modification systems and CRISPR-Cas systems) were reported (Stucken et al., 2013). *M. aeruginosa* is a unicellular cyanobacterium and usually forms colonial particle with an amorphous mucilage or sheath in wild environment (Vinh et al., 2012). This effective physical barrier makes their contact with foreign genetic material much more difficult. Besides, many genes for restriction modification (R-M) systems were characterized in both NIES-843 (Kaneko et al., 2007) and PCC 7806 (Frangeul et al., 2008) genomes.

The CRISPR-Cas systems of some cyanobacteria have been reported and they could play an important role in defending invasion of foreign DNA elements (Scholz et al., 2013). The CRISPR-Cas systems in *M. aeruginosa* are characterized here and our results show that each genome of *M. aeruginosa* containing at least one CRISPR locus reveals a widespread distribution among this species. Complete CRISPR-Cas system comprised of CRISPR locus and *cas* genes nearby is existing in 12 genomes, except for strain T1-4 and PCC 9806. These two strains only have CRISPR loci but no *cas* gene is found in the genomes. The absence of *cas* gene in these two strains is unlikely due to sequencing/assembly errors since no proto-spacers were shown in T1-4 and PCC 9806. In support of this suggestion, *Lactobacillus brevis* ATCC 367 with completely sequenced genome has no *cas* gene while CRISPR is present (Horvath et al., 2009).

In addition to the previously reported subtype I-D cas gene sets (Kuno et al., 2012), *M. aeruginosa* as a species contains the most diversified CRISPR DR and CAS types in bacteria when all the strains are considered collectively. CRISPR-Cas system in *M. aeruginosa* displays high heterogeneity between strains on both CAS subtype and CRISPR array sequence. There are no two identical CRISPR-Cas systems in the different strains of *M. aeruginosa*. The high degree of heterogeneity CRISPR-Cas system demonstrates the evolutionary dynamics of *M. aeruginosa*. It also implies great capacity for CRISPR-Cas systems to keep genome stable by restricting high-frequency HGT. It is interesting to note that, because the CRISPR units are not present in the core genes of *M. aeruginosa*, the diversified CRISPR systems were likely a result of HGT itself. In *M. aeruginosa*, there are total seven types of CRISPR direct repeat (DR) and each of them has a clear, stable secondary RNA structures, indicating their closely association to strain-specific habit.

Although highly conserved Cas1 and Cas2 proteins are considered as the prime candidates for new spacer acquisition (Barrangou et al., 2007; Brouns et al., 2008), Cai et al. revealed that approximately 33% of cyanobacterial genomes lacked these two genes and they had other *cas* gene operons in genomes (Cai et al., 2013). Our work also observed the absence of *cas1* and *cas2* genes in TAIHU98 and PCC 9432. These strains may have either lost the function for novel spacer acquisition or have a different mechanism for acquisition of new spacers. The presence of an additional CRISPR locus present in these genomes is interesting and needs further investigate.

Analyzing the diversity and origin of the spacers provides means to explore functional roles of CRISPR-Cas systems in corresponding *M. aeruginosa* genomes. The proto-spacers identified by homology searches reveal a history of resistance to known bacteriophage and plasmids associated with *M. aeruginosa*, but also the ability to target much more unknown exogenous genetic material in the natural environment.

## Conclusion

Genomes of *M. aeruginosa*, which are found in diverse freshwater ecological environments, show highly plasticity with stable core genes. Our study shows that *M. aeruginosa* can retain the genomic variations that are beneficial for survival and proliferation and have defense systems to prevent harmful invasion of foreign elements. The CRISPR-Cas systems of *M. aeruginosa* are important in keeping genomic stability and shaping the genomes of these species. Thus, maintaining genomic stability and modulating genomic plasticity are key features of *M. aeruginosa*, which lead them to adapt and survive in various habits.

### Conflict of interest statement

The authors declare that the research was conducted in the absence of any commercial or financial relationships that could be construed as a potential conflict of interest.
