# Supplementary material for: Comparative genomics reveals diversified CRISPR-Cas systems of globally distributed Microcystis aeruginosa, a freshwater bloom-forming cyanobacterium
Source: Front Microbiol. 2015 May 12;6:394. doi: 10.3389/fmicb.2015.00394 (PMC4428289; doi:10.3389/fmicb.2015.00394)
Supplement: Supplementary file 5 [file Presentation1.PDF]

Supplementary Material

Comparative genomics reveals diversified CRISPR-Cas systems of globally distributed *Microcystis aeruginosa*, a freshwater bloom-forming cyanobacterium

Chen Yang[1,2], Feibi Lin[1,2], Qi Li[1,2], Tao Li[1]*, Jindong Zhao[1,3]*

[1] Key Laboratory of Algal Biology, Institute of Hydrobiology, Chinese Academy of Science, Wuhan, China
[2] University of Chinese Academy of Sciences, Beijing, China
[3] College of Life Science, Peking University, Beijing, China

*** Correspondence:**

Dr. Tao Li, Key Laboratory of Algal Biology, Institute of Hydrobiology, Chinese Academy of Science, No. 7 Donghu South Road, Wuhan, 430072, China.
E-mail: litao@ihb.ac.cn
Prof. Jindong Zhao, Key Laboratory of Algal Biology, Institute of Hydrobiology, Chinese Academy of Science, No. 7 Donghu South Road, Wuhan, 430072, China.
E-mail: jzhao@ihb.ac.cn

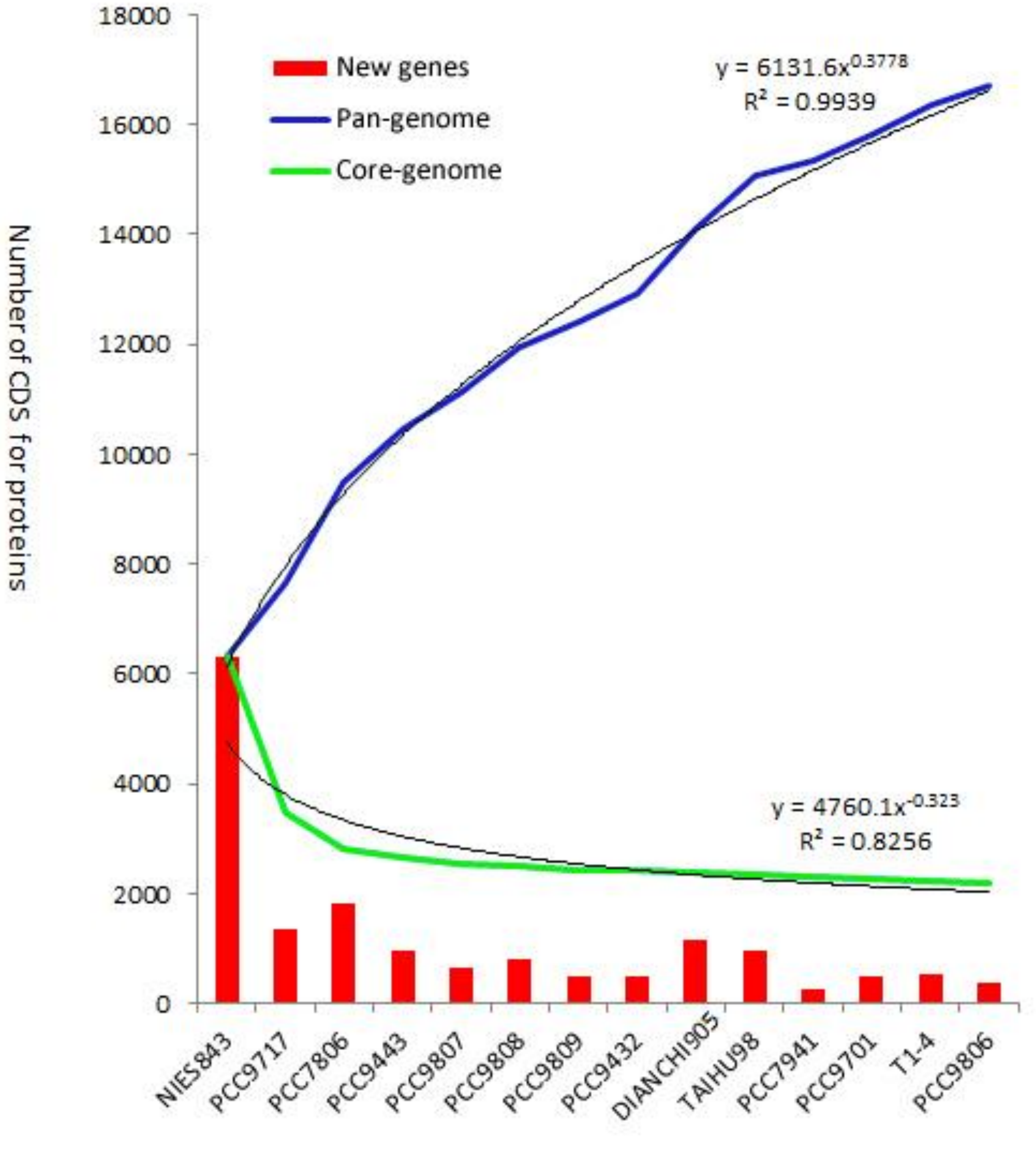

Figure S1. Estimation of core- and pan-genome in *M. aeruginosa.* A pan- and core-genome plot of 14 *M. aeruginosa* strains. The green and blue lines show the core- and pan-genomes as more and more genomes are added into calculation, while the red columns indicate the amount of novel gene families.

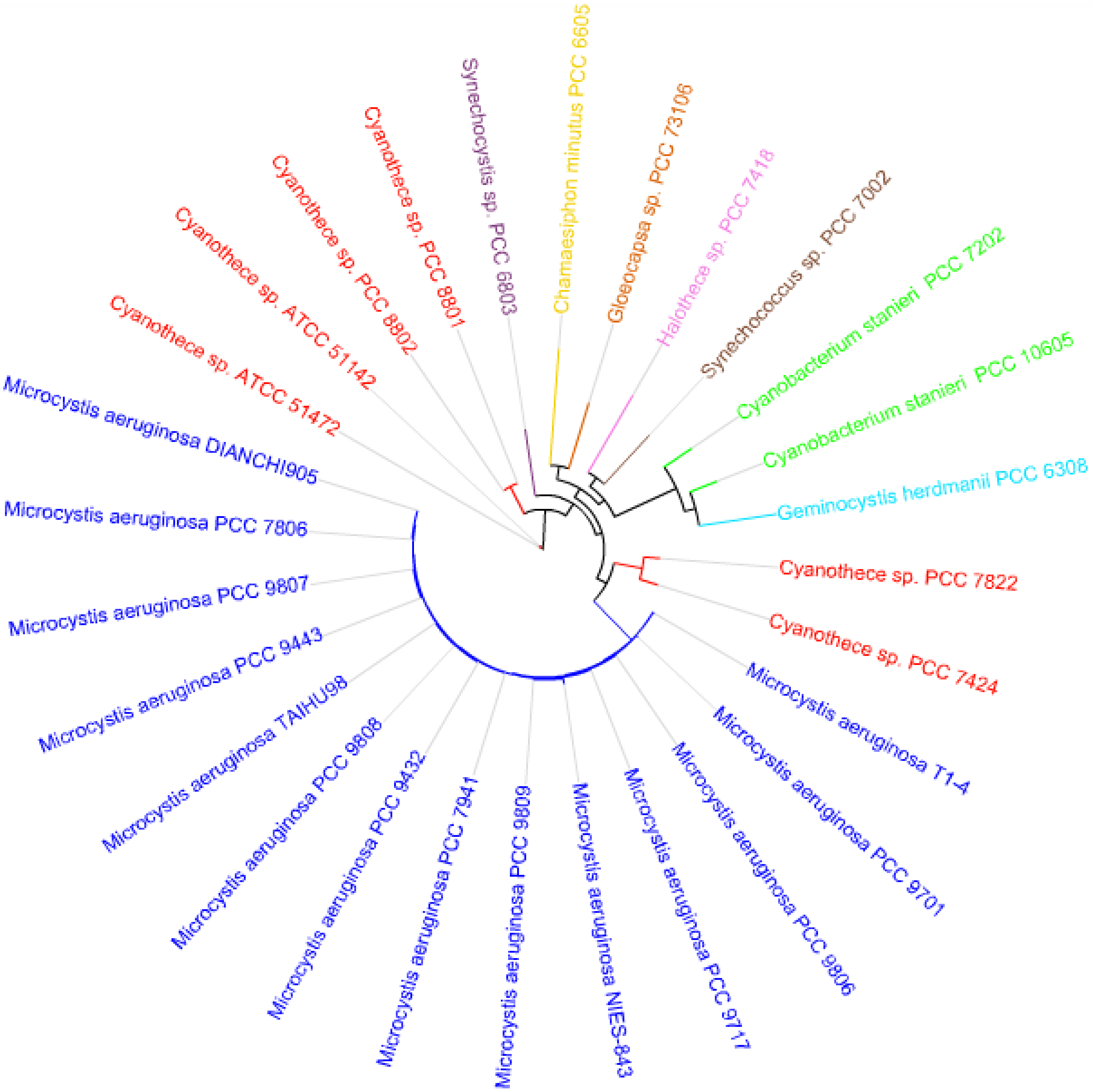

Figure S2. Species tree of cyanobacterial genomes used in this paper. An unrooted maximum likelihood genome tree based on 31 concatenated conserved proteins. Branches are colored according to genome species (Deep Blue: *Microcystis aerugionsa* ;Red: *Cyanothece* sp.; Purple: *Synechocystis* sp.; Brown: *Syechococcus* sp.; Yellow: *Chamaesiphon minutes*; Orange: *Gloeocapsa* sp; Pink: *Halothece* sp.; Green: *Cyanobacterium stanieri*; Light Blue: *Geminocystis herdmanii*).

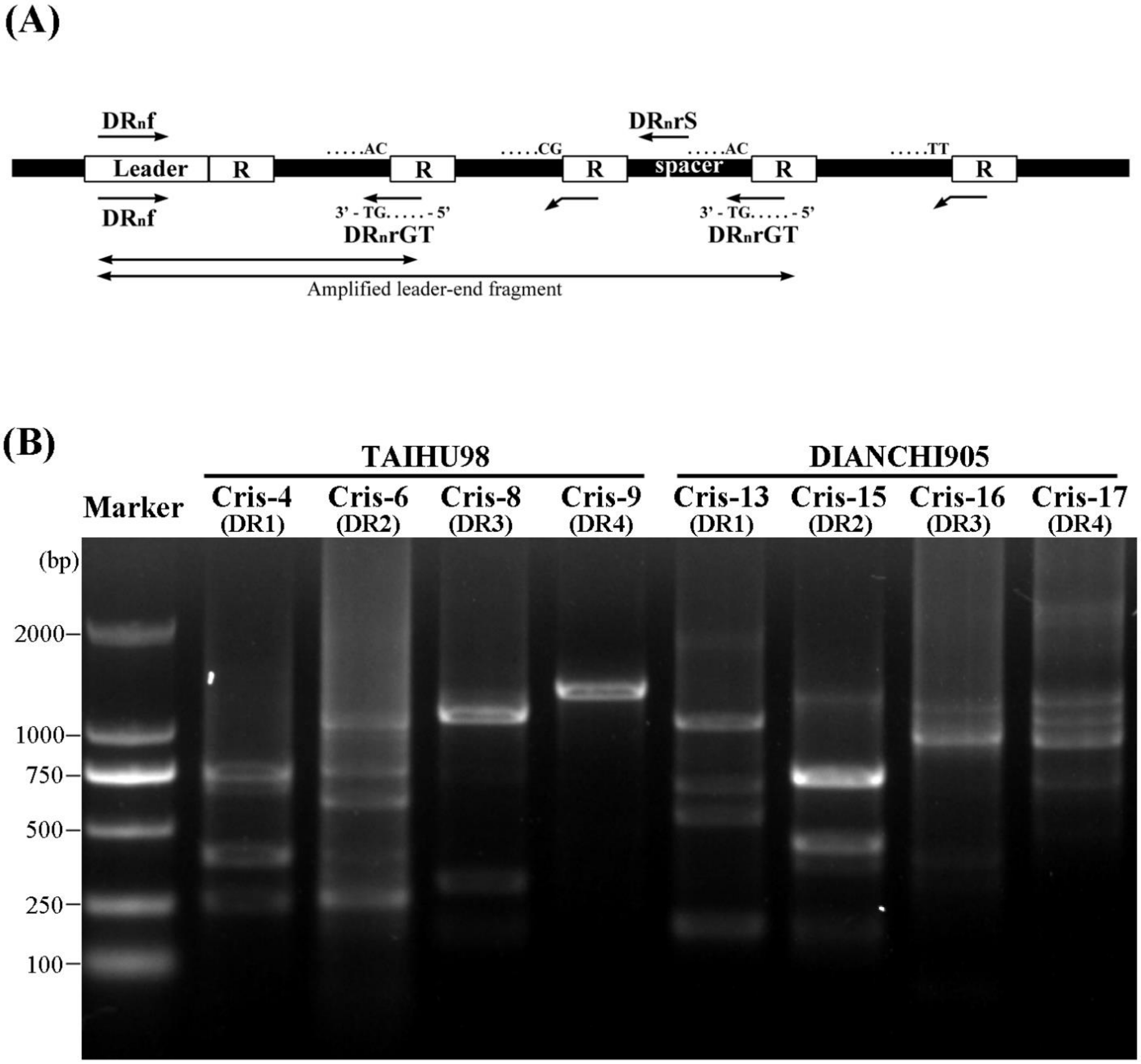

Figure S3. Amplication of leader-end CRISPR fragments. (A) Schematic diagram of primers designed for CRISPR fragments amplication and sequencing. DRnrNN was designed with two additonal nucleotides, for example GT, thereby prferentially annealing to a limited number of specific spacer-repeat units. For different DR types and different strains, the reverse primers have different two nucleotiders. (B) PCR products of CRISPR fragments in TAIHU98 (Cris-4 contains DR1, Cris-6 contains DR2, Cris-8 contains DR3, Cris-9 contains DR4) and DIANCHI905 (Cris-13 contains DR1, Cris-15 contains DR2, Cris-16 contains DR3, Cris-17 contains DR4).

Table S1. Primers used for the varification of CRISPR array.

Strain	CRISRP No.	CRISPR locus	F primer	Sequence(5'-3')	R primer	Sequence(5'-3')	Purpose	Sequencing length	Alignment identity
TAIHU98	Cris-4	NZ_ANKQ01000003.1	98DR1f	GTCTATTGAAACAATCGGCGGG	98DR1rGC	GGTTTAAGATTAATTGGAACGC	repeat-based PCR, CRISPR amplification	-	-
					98DR1rS	CCATTAGGGTTCGGAGACGG	spacer-based PCR, CRISPR sequencing	803	100
	Cris-6	NZ_ANKQ01000001.1	98DR2f	AGGCTTTGAGCCTGATGACC	98DR2rGT	GCTTCACGAATTGGAAGCAAGGT	repeat-based PCR, CRISPR amplification	-	-
					98DR2rS	GCCGTCCTAGGTAACCTCCA	spacer-based PCR, CRISPR sequencing	930	99.8
	Cris-8	NZ_ANKQ01000002.1	98DR3f	CAGTCAAGGGGAAAGCCGAT	98DR3rGC	TTCTCTAGCGAGTAGAGAGCG	repeat-based PCR, CRISPR amplification	-	-
					98DR3rS	CGAGACCAATACCCAGCGTT	spacer-based PCR, CRISPR sequencing	774	99.7
	Cris-9	NZ_ANKQ01000002.1	98DR4f	GCTTTTGCCCCACTGTTTCC	98DR4rTT	AATAGGATTAGTTGGAAACTT	repeat-based PCR, CRISPR amplification	-	-
					98DR4rS	CCCCGATTCTCTGGTTGAGG	spacer-based PCR, CRISPR sequencing	665	100
DIANCHI905	Cris-13	AOCI01000069.1	905DR1f	GTGGGGAGAAGGAACAAGGG	905DR1rAG	ACCCTATTAGGGATTGAAACAG	repeat-based PCR, CRISPR amplification	-	-
					905DR1rS	GCCCGGAGACAAATCTGGAA	spacer-based PCR, CRISPR sequencing	816	100
	Cris-15	AOCI01000017.1	905DR2f	TTCAACAGCCTTGAAAAGACCT	905DR2rTT	TGAAGCGTATGAATGGAAACTT	repeat-based PCR, CRISPR amplification	-	-
					905DR2rS	CGTCGATGCCTTGTCCAGTA	spacer-based PCR, CRISPR sequencing	949	99.6
	Cris-16	AOCI01000034.1	905DR3f	ACAGGGGTGGGAGCAGATAA	905DR3rTG	TTCTCTAGCGAGTAGAGAGATG	repeat-based PCR, CRISPR amplification	-	-
					905DR3rS	ATCCCCTTGAGGTGCGATTG	spacer-based PCR, CRISPR sequencing	597	100
	Cris-17	AOCI01000163.1	905DR4f	CACATCGAGAGGCGGAAACT	905DR4rTC	TTTGACCTAATAGGTAAGGTC	repeat-based PCR, CRISPR amplification	-	-
					905DR4rS	ATAGGTAAGGCCAGCCGTGA	spacer-based PCR, CRISPR sequencing	1069	99.8

Table S2. Occurrence of CAS subtypes in the proximity of the seven direct repeats. Associations are indicted by check marks (√), while a dash (-) means DR were not detected in this CRISPR-Cas system.

CRISPR-Cas type	DR type						
	1	2	3	4	5	6	7
Subtype I-A	-	-	-	-	-	-	√
Subtype I-D	√	-	-	-	-	-	-
Subtype III-A	-	-	-	-	-	√	-
Subtype III-B	-	√	√	√	-	-	-
